# Maxillary Abscess

**Published:** 1849-04

**Authors:** Samuel Griffith


### MAXILLARY ABSCESS.

BY SAMUEL GRIFFITH.

I AM induced to present this case to the public, to show the inexperienced that they should not pass over what they may suppose to be merely slight inflammation of the jaw from a diseased tooth.

In the fall of 1827, Miss D——, a lady of Louisville, Ky., (who had been a patient of mine for several years,) in my absence from the city, called on a gentleman of the profession to have a molar tooth extracted; (the second molar of the upper jaw;) three or four days afterwards, in consequence of great swelling of the face, accompanied with intense pain, she requested him to examine the jaw, to see what was the matter. He examined the mouth, and told her it was only a slight inflammation, which would be well in a short time. A few days after this, I returned home, when she called on me, told what had happened in my absence, and said the pain seemed to be more violent than ever, and she thought she would go crazy unless she could be relieved. Her face was very much swollen, and the cheek over the antrum flushed to a considerable extent. I examined the mouth, and, as might have been expected, found the gums swollen and inflamed. I introduced a probe through the cavity left by the extracted tooth; it passed readily through the antrum, and, upon being withdrawn, was followed by a slight discharge of matter. I then told my patient what was the matter, and that she must not be discouraged, notwithstanding the disease might be a little obstinate, and probably require some months for its cure; that she would be entirely relieved, provided she strictly followed my directions.

I prepared a wash consisting of eight drops of the oil of creosote to one ounce of water, held in solution with a small quantity of gum arabic, and desired that this should be thrown into the antrum twice a day, by means of a small syringe, a pledget to be used in the interval to prevent the orifice from closing. At the end of two weeks she called on me for a fresh supply of the wash, suffering but little pain comparatively, the swelling much reduced, and a copious discharge of fetid matter issuing from the antrum. Upon introducing the probe, I found it met with much less resistance, and was accompanied with much less pain than in the first instance. I directed her to continue the wash as before, strengthening it by the addition of two drops of the creosote.

At the end of two weeks my patient called again, in much better spirits; the swelling had gradually diminished, the gums assuming a more healthy character, but the discharge was rather darker, and not so abundant. I then changed the wash to a weak solution of sulphate of zinc and myrrh. In about three weeks a great improvement was manifest. I continued this treatment, gradually increasing the strength of the injection, for about two months, when the cure was complete.

In some cases I have been compelled to use the nitrate of silver in solution once or twice, and then return to the former wash. The pledget should never be removed until the discharge has nearly or quite ceased, except to inject the parts.

LOUISVILLE, KY., February 26, 1849.

